# Hydroxychloroquine (HCQ) decreases the benefit of anti-PD-1 immune checkpoint blockade in tumor immunotherapy

**DOI:** 10.1371/journal.pone.0251731

**Published:** 2021-06-28

**Authors:** Janna Krueger, Francois Santinon, Alexandra Kazanova, Mark E. Issa, Bruno Larrivee, Richard Kremer, Catalin Milhalcioiu, Christopher E. Rudd

**Affiliations:** 1 Division of Immuno-Oncology, Research Center Maisonneuve-Rosemont Hospital (CR-HMR) Montreal, Quebec, Canada; 2 Department of Microbiology, Infection and Immunology, Universite de Montreal, Montreal, Quebec, Canada; 3 Department of Ophthalmology, Universite de Montreal, Montreal, Quebec, Canada; 4 Division of Experimental Medicine, Department of Medicine, McGill University Health Center, Montreal, Canada; 5 Division of Medical Oncology, Department of Medicine, McGill University Health Center, Montreal, Canada; Emory University Hospital Midtown, UNITED STATES

## Abstract

Immunotherapy using checkpoint blockade (ICB) with antibodies such as anti-PD-1 has revolutionised the treatment of many cancers. Despite its use to treat COVID-19 patients and autoimmune diseases such as systemic lupus erythematosus and rheumatoid arthritis, the effect of hydroxychloroquine (HCQ) on cancer immunotherapy has not been examined. In this study, remarkably, we find that HCQ alone, or in combination with azithromycin (AZ), at doses used to treat patients, decreased the therapeutic benefit of anti-PD-1 in cancer immunotherapy. No deleterious effect was seen on untreated tumors. Mechanistically, HCQ and HCQ/AZ inhibited PD-L1 expression on tumor cells, while specifically targeting the anti-PD-1 induced increase in progenitor CD8^+^CD44^+^PD-1^+^TCF1^+^ tumor infiltrating T cells (TILs) and the generation of CD8^+^CD44^+^PD-1^+^ effectors. Surprisingly, it also impaired the appearance of a subset of terminally exhausted CD8^+^ TILs. No effect was seen on the presence of CD4^+^ T cells, FoxP3^+^ regulatory T cells (Tregs), thymic subsets, B cells, antibody production, myeloid cells, or the vasculature of mice. This study indicates for the first time that HCQ and HCQ/AZ negatively impact the ability of anti-PD-1 checkpoint blockade to promote tumor rejection.

## Introduction

The anti-malarial 4-aminoquinoline drugs chloroquine (CQ) and hydroxychloroquine (HCQ) as well as the antibiotic azithromycin (AZ) have gained much attention as potential therapies. The parent compound chloroquine (CQ) was originally reported to inhibit SARS-Cov-1 coronavirus infection [1] and *in vitro* studies have shown activity against SARS-CoV-2 [2, 3]. The exact mechanism of action of CQ and HCQ is unknown although these drugs increase the pH of endosomes that the virus uses for cell entry and can interfere with the glycosylation [4]. Other mechanisms have also been proposed [5]. CQ has a half-life of 20–60 days and can accumulate at higher levels in metabolically active tissues [6]. Similarly, a macrolide antibiotic AZ can synergise with HCQ to block viral entry into cells and decrease viral replication [7].

Aside from the treatment of malaria, HCQ has been used widely over the past decade at lower concentrations to treat auto-immune diseases such as systemic lupus erythematosus and rheumatoid arthritis where it can reduce inflammation [8, 9]. More recently, amid controversy, it has and continues to be used to treat coronavirus disease 2019 (COVID-19) [10, 11]. The severe acute respiratory syndrome coronavirus 2 (SARS-CoV-2/2019-nCoV) poses a serious threat to global public health. An initial non-randomised trial showed remarkable efficacy in combination with AZ in clearing SARS-Cov-2 [12]. Other studies have produced conflicting results [13–23]. However, adverse cardiovascular effects may predispose certain patient groups to ventricular arrhythmias [24, 25]. The efficacy may vary with the dose used in the various studies. One recent study from the multi-center COVID-19 RISK and Treatments (CORIST) Collaboration, using 400–600 mg of HCQ per day found that treatment was associated with reduced mortality [26]. Another recent study from Belgium using similar doses also reported reduced mortality [27]. The doses and onset of treatment in these studies differed from the UK RECOVERY trial where 1600 mg on the first day and 800 mg on days 2–9 were given [28]. Overall, there is an interest whether HCQ might be more effective if used early in infection, before the need for hospitalisation. Many countries continue to use HCQ to treat COVID-19 health workers who are suspected or confirmed of infection [29].

In terms of the immune response, HCQ and CQ have been reported to have mixed and contradictory effects on the immune system. One report showed that CQ enhances human CD8^+^ T cell responses [30], while another showed that HCQ inhibits CD4^+^ T cell activation [31]. HCQ is well known to inhibit autophagy [32], may reduce the efficacy of antigen-presentation [8, 33] and decrease the production of proinflammatory Th17-related cytokines [34]. Another report showed that HCQ enhanced the function of suppressive regulatory T cells (Tregs) [9].

In a related context, the past 15 years have witnessed a revolution in the application of immunotherapy for the treatment of cancer. Immune checkpoint blockade (ICB) uses monoclonal antibodies that block the binding of inhibitory receptors (IRs) on T cells to their natural ligands often expressed on cancer cells. The blockade of cytotoxic T-lymphocyte–associated antigen 4 (*CTLA*-*4*) and programmed death 1 (PD-1) or the PD-1 ligand, PD-L1 have achieved survival rates of 20–30% in treating cancers such as non-small cell lung carcinoma (NSCLC), melanoma, kidney, and bladder cancer [35, 36]. The tumor microenvironment (TME) can alter the make-up and function of TILs [37].

With the use of HCQ in treating autoimmunity and COVID-19, a percentage of these patients will be cancer patients on ICB. This raises an important question as to the best therapeutic approach for COVID-19 patients, one that limits viral spread, while allowing for the benefit of checkpoint immunotherapy in promoting reactivity against tumor neo-antigens. This has been further complicated by reports that HCQ can reverse the drug sequestration in lysosomes [38] and enhance chemo-sensitization in cancer patients [38]. Phase II trial studies showed that HCQ is effective in treating patients with recurrent oligometastatic prostate cancer by promoting cell death in cancer cells. In breast cancer, autophagy has been reported to be tumour-suppressive [39, 40], while in other cases, can promote tumors [41, 42]. In a similar vein, AZ can inhibit primary antibody responses, and recall responses on bacterial infections [43]. Although these previous studies emphasized effects on the gut microbiota [44, 45], it is noteworthy that AZ and ciprofloxacin (CPX) also act as lipophilic weak bases where they affect intracellular organelles similar to CQ and HCQ [46]. HCQ has been reported to potentiate the effects of anti-PD-1 against B16 melanoma cells in C57BL/6 mice via the inhibition of palmitoyl-protein thioesterase 1 (PPT1) [47].

Given the urgent clinical context, there is a pressing need to assess the effects of both HCQ and AZ on the immune checkpoint response against cancer. In this study, we show that HCQ and AZ, alone or in combination, impaired the ability of anti-PD-1 therapy to promote the elimination of the B16 melanoma. We further show that HCQ or HCQ/AZ combined therapy selectively inhibits the appearance of self-renewing CD8^+^ PD-1^+^TCF1^+^ TILs and CD8^+^ PD-1^+^ TOX^+^ effector T cells. Our study indicates that HCQ negatively affects anti-PD-1 immune checkpoint blockade in the promotion of tumor rejection.

## Results

### HCQ and AZ reduce the efficacy of anti-PD-1 therapy

**Fig 1:**
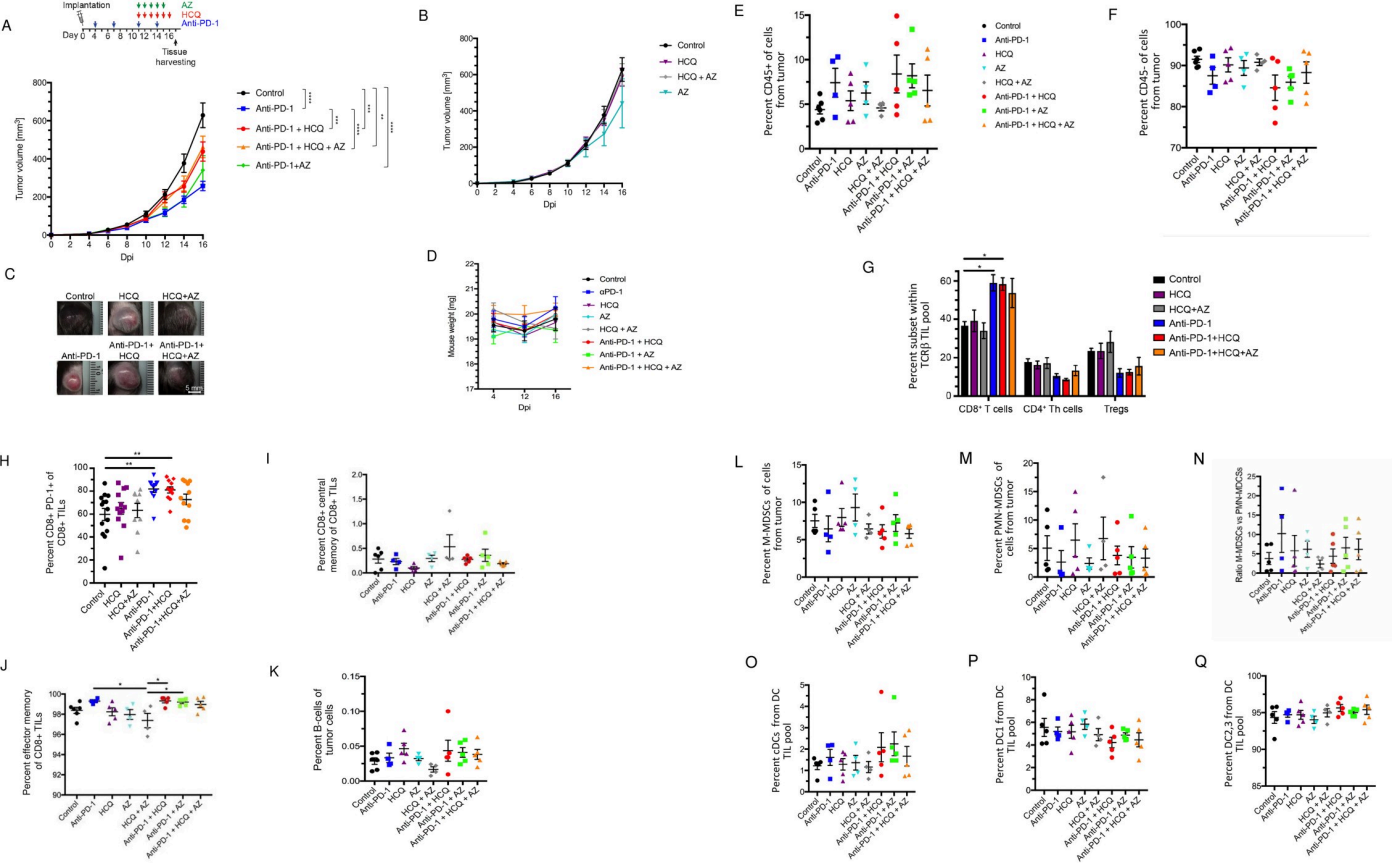
Hydroxychloroquine and azithromycin reverse partially immune checkpoint blockade in cancer therapy. C57BL/6 mice were implanted intradermally with B16-PD-L1 tumor cells. Following 1 week of anti-PD-1 treatment, HCQ with or without AZ were injected daily before tumors were harvested at day 17 post implantation. **Panel A:** Tumor volumes in response to anti-PD-1 plus HCQ and AZ. **Panel B:** Tumor volumes in mice treated only with HCQ and AZ. **Panel C:** Examples of tumors from mice treated with HCQ, AZ or anti-PD-1 plus HCQ and AZ. **Panel D:** Percentage of mouse weight loss over time. **Panel E:** Percentage of total immune cell infiltrate (CD45^+^ cells). **Panel F:** Percentage of CD45^-^ cells in tumors. **Panel G:** Percentage of CD8^+^, CD4^+^ T helper and regulatory T cells (Tregs) of total TCRb^+^ cells in tumor. **Panel H:** Percentage of PD-1^+^ CD8^+^ TILs. **Panel I:** The effect of therapy on CD8^+^ central memory TILs. **Panel J:** The effect of therapy on CD8^+^ effector memory TILs. **Panel K:** The effect of therapy on B cell TILs. **Panel L:** The effect of therapy on the percentage representation of M-MDSC TILs. **Panel M:** The effect of therapy on the percentage representation of PMN-MDSC TILs. **Panel N:** Ratio of M-MDSC TILs relative to PMN-MDSC TILs. **Panel O:** The effect of therapy on the percentage representation of cDC TILs. **Panel P:** The effect of therapy on the percentage representation of DC1 TILsc. **Panel Q:** The effect of therapy on the percentage representation of DC2 and 3 TILs.

To address this issue, we assessed whether HCQ or AZ was beneficial, or harmful to tumor immunotherapy. Mice were implanted with B16-PD-L1 melanoma cells intradermally into C57BL/6J mice 4 days before the injection of anti-PD-1 alone (200ug/mouse), or in conjunction with HCQ or AZ at days 4, 7, 11 and 14. Tumors were harvested on day 17. The dosing with HCQ and AZ was similar as used in clinical trials to treat SARS-CoV-2 infection [12]. **Fig 1A** shows that the growth curve in which tumour volume increased from 100mm^3^ on day 10 to 610mm^3^ from day 16 (n = 14). Anti-PD-1 reduced the growth of tumor as early as day 12 (i.e. 100 vs 210 mm^3^) until day 16 (i.e. 220 vs 610mm^3^). Importantly, HCQ impaired the anti-PD-1 reduction in tumor growth as seen on day 16 (i.e. 425mm^3^ for HCQ/anti-PD-1 vs. 210mm^3^ for anti-PD-1). Injection of AZ alone showed a trend in reducing the beneficial effect of anti-PD-1 (375mm^3^ vs 210mm^3^ for anti-PD-1), although this did not achieve statistical significance. The combination of HCQ and AZ reversed the benefits of anti-PD-1 to the same extent as HCQ alone (430mm^3^ vs 220mm^3^ for anti-PD-1) (also see spider graphs in **S1** and **S2 Figs**). Interestingly, neither HCQ nor AZ affected tumor growth in the absence of anti-PD-1 (**Fig 1B**). Examples of control and treated tumors are shown in **Fig 1C**. Neither drug treatment resulted in major loss of mouse weight (**Fig 1D**), while scatter plot analysis showed a correlation between tumor volume and weight in reducing tumor size in response to anti-PD-1, HCQ and HCQ/AZ (p = 0.025) (**S3 Fig**). HCQ did not directly affect the growth of *in vitro* cultured B16 cells (**S4 Fig**). These data showed that HCQ and HCQ/AZ impaired the ability of anti-PD-1 to promote tumor regression.

We next examined the composition of TILs in tumors where anti-PD-1 showed a trend in increasing CD45^+^ TILs (**Fig 1E**). Neither HCQ nor AZ affected this overall increase. Conversely, anti-PD-1 showed a trend in reducing the percentage of CD45^-^ cells, although again, this was not statistically significant (**Fig 1F**). In examining specific subsets, we showed that anti-PD-1 caused a significant increase in the overall presence of CD8^+^ TCRβ^+^ TILs, which was unaffected by HCQ or AZ (**Fig 1G**). The same treatment showed a trend in decreasing the percentage of CD4^+^ Thelper cells and CD4^+^ FoxP3^+^ TILs. Neither AZ, nor HCQ affected this trend.

Within the CD8 TIL compartment, anti-PD-1 therapy increased the representation of CD8^+^ CD44^+^ PD-1^+^ cells (**Fig 1H**), while the presence of HCQ and AZ interfered with the ability of anti-PD-1 to achieve statistical significance relative to untreated mice. By contrast, no differences were noted in CD8^+^ central memory TILs (**Fig 1I**), CD8^+^ effector memory T cells (**Fig 1J**) or B cells (**Fig 1K**).

In terms of myeloid cells, cancer-driven emergency myelopoiesis give rises to myeloid-derived suppressor cells (MDSCs) that express PD-1 [48, 49]. Anti-PD-1 therapy showed a trend in decreasing the presence of suppressive M-MDSCs (**Fig 1L**) and PMN-MDSCs (**Fig 1M**) as well as in increasing the ratio of M-MDSCs to PMN-MDSCs (**Fig 1N**), as reported [48]. HCQ nor AZ had a statistically irrelevant effect on this trend. Similarly, anti-PD-1 showed a trend in increasing the presence of cDC TILs which was unaffected by HCQ or HCQ/AZ (**Fig 1O**). No obvious effect of therapies on the presence of DC1 (**Fig 1P**) or DC2 and 3 TILs was observed (**Fig 1Q**).

**Fig 2:**
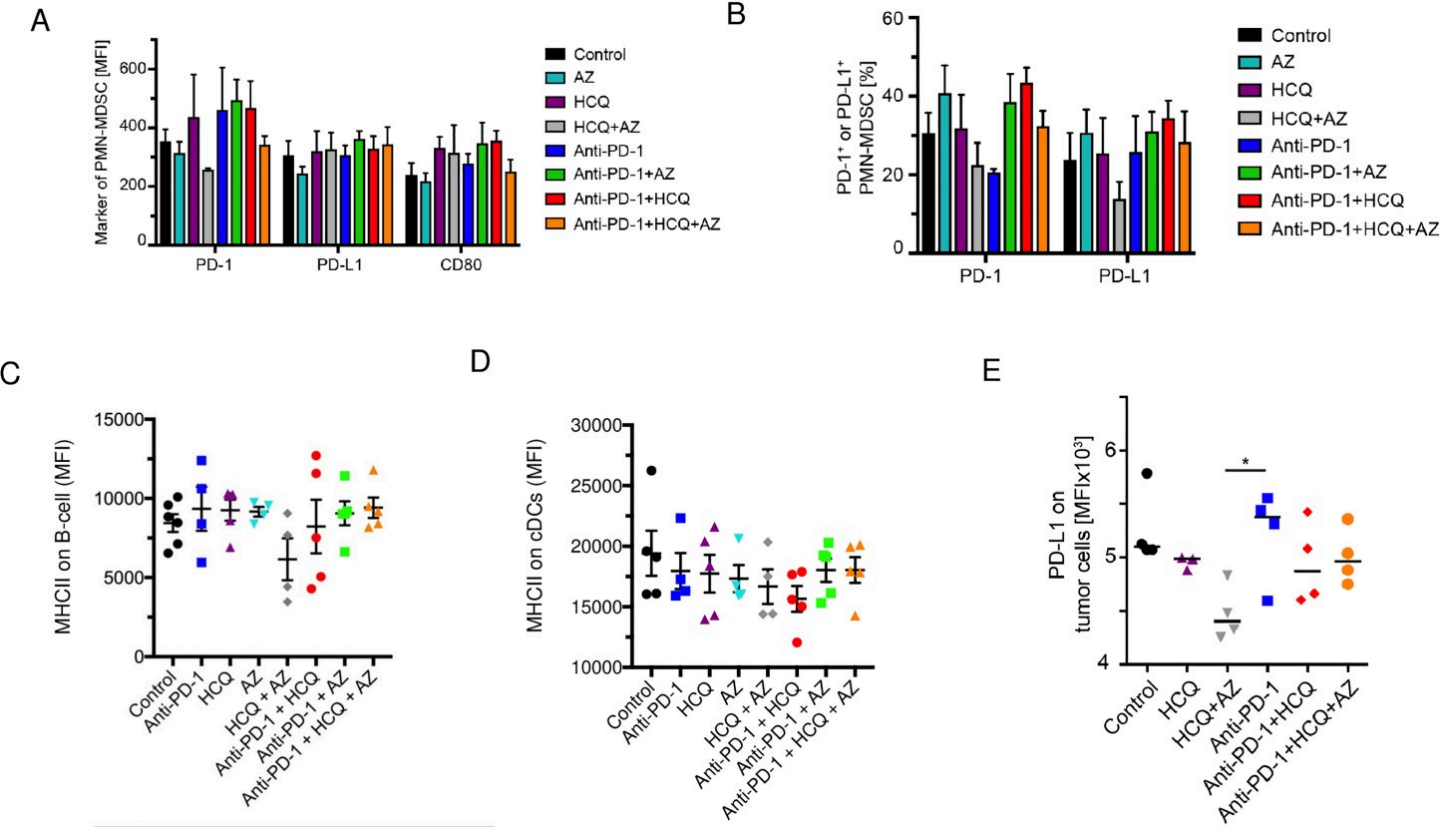
The effect of HCQ and AZ on the expression of PD1, PD-L1 and CD80 on TIL subsets. **Panel A:** Histogram showing the medium expression of PD1, PD-L1 and CD80 on PMN-MDSC TILs. **Panel B:** Histogram showing the percent positive PMN-MDSC TILs expressing PD1 and PD-L1. **Panel C:** The effect of therapy on MHC class II expression (MFI) on B cells. **Panel D:** The effect of therapy on MHC class II expression (MFI) on cDCs. **Panel E:** The effect of HCQ and HCQ/AZ on the anti-PD-1 induced increase in PDL-1 expression in vitro cultured B16 cells.

We next examined the surface expression of PD-1, PD-L1 and CD80 receptors on different TILs (**Fig 2**). Anti-PD-1 treatment showed a trend in increasing PD-1 expression on PMN-MDSCs (**Fig 2A**). Neither HCQ nor AZ monotherapy in combination with anti-PD-1 affected this trend, although the combination of HCQ/AZ reduced expression to control levels. No effect on the expression of PD-L1 or CD80 was observed. Anti-PD-1 increased the percentage of PD-1 expressing PMN-MDSCs which was unaffected by co-injection of HCQ or AZ in mice (**Fig 2B**). The percentage of PMN-MDSCs TILs expressing PD-L1 was unaffected by anti-PD-1 or anti-PD-1 with HCQ or AZ (**Fig 2B**). Similarly, no effect was observed on the expression of class 2 major histocompatibility antigens (MHC) on B cells (**Fig 2C**) or cDCs (**Fig 2D**). Likewise, anti-PD-1 treatment and HCQ or HCQ plus AZ had no appreciable effects on the MFI of PD-L1 expression on CD45^-^ CD31^-^ cells, which likely represent B16 tumor cells, relative to the control (**Fig 2E**). Further, no effect on the expression of other key ligands on myeloid, B cells or on the B16 melanoma cells was observed. We also did not observe changes in the presence of IgM or IgG antibodies against PD-L1 in mice treated with anti-PD1, alone, or in combination with HCQ/AZ (**S4 Fig**). Index value was calculated by normalizing B16-IgG and B16-IgM MFI of the sample to the average of tumor-free mice (**S4D–S4F Fig**). We also did not observe effects on the composition of thymic subsets (**S5 Fig**).

### HCQ and AZ impairs anti-PD-1 induction of progenitor CD8^+^ PD-1^+^ TCF1^+^ TILs

**Fig 3:**
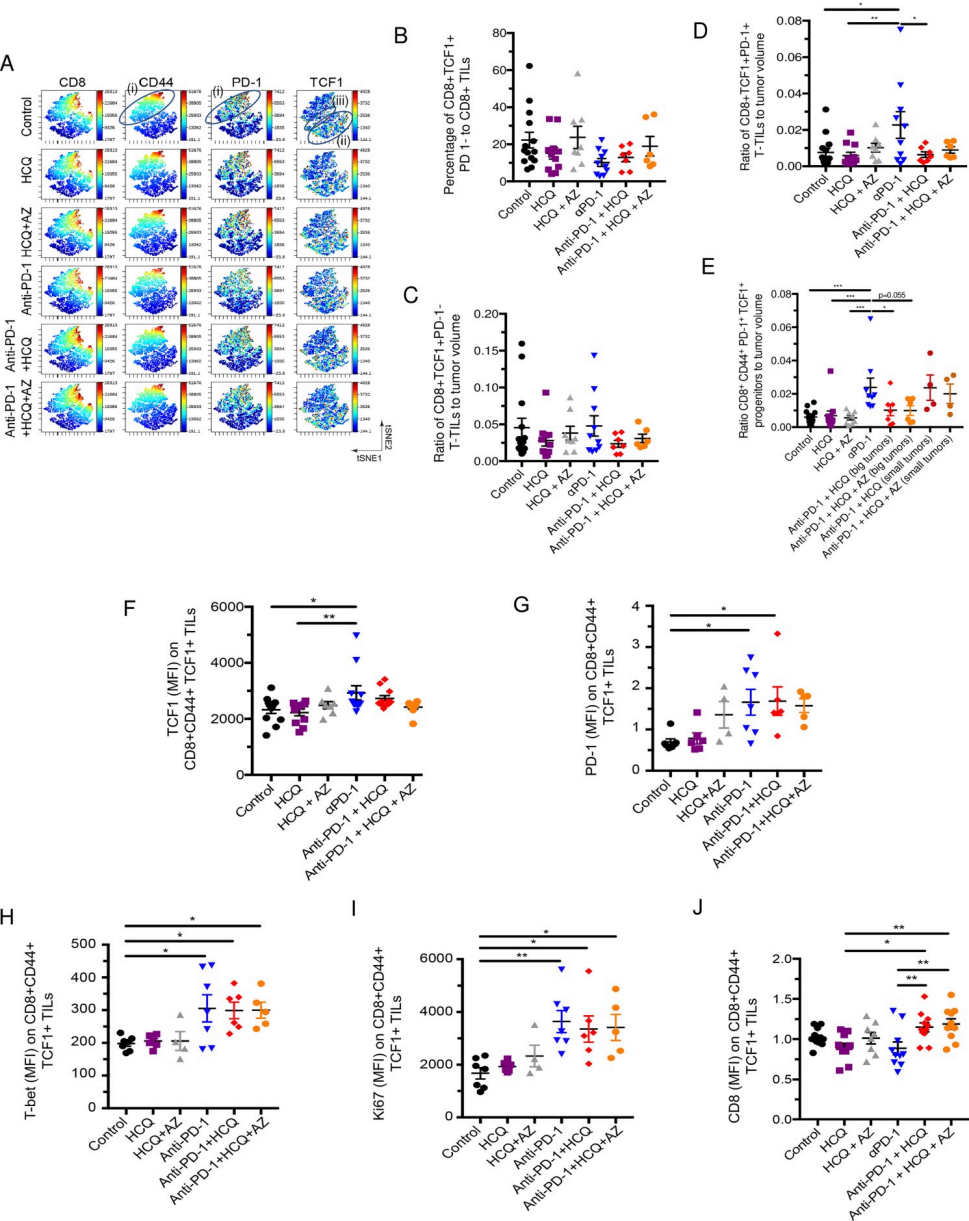
The effect of HCQ and AZ on the presence of CD8^+^TCF1^+^ progenitor TILs. Mice were implanted with B16 tumours as described in Fig 1. **Panel A:** viSNE profiles of total CD8^+^ TILs. The figure shows the presence of the upper cluster of TILs expressing moderate to high levels of CD8, PD-1 and CD44 (island (i)), a second island (ii) expressing moderate levels of TCF-1^+^ with no PD-1; and island (iii) that co-expresses PD-1^+^ and TCF-1^+^. **Panel B**: Percentage of CD8^+^ TCF-1^+^ PD-1^-^ TILs as a percent of total CD8+ TILs. **Panel C:** Ratio of CD8^+^ CD44^+^TCF-1^+^ PD-1^-^ TILs relative to tumor volume. **Panel D:** Ratio of CD8^+^ TCF-1^+^ PD-1^+^ TILs relative to tumor volume. **Panel E:** Ratio of CD8^+^ CD44^+^ PD-1^+^TCF-1^+^ progenitor TILs relative to tumor volume. A comparison of tumor volumes in mice where HCQ blocked the response to anti-PD-1 (big tumors) and mice where HCQ failed to block the response to anti-PD-1 (small tumors). **Panel F:** MFI of TCF-1 expression CD8^+^ CD44^+^TCF-1^+^ PD-1^+^ TILs in response to anti-PD-1 alone or in combination with HCQ or HCQ/AZ. **Panel G:** MFI of PD-1 expression CD8^+^ CD44^+^TCF-1^+^ PD-1^+^ TILs in response to anti-PD-1 alone or in combination with HCQ or HCQ/AZ. **Panel H:** MFI of T-bet expression CD8^+^ CD44^+^TCF-1^+^ PD-1^+^ TILs in response to anti-PD-1 alone or in combination with HCQ or HCQ/AZ. **Panel I:** MFI of Ki67 expression CD8^+^ CD44^+^TCF-1^+^ PD-1^+^ TILs in response to anti-PD-1 alone or in combination with HCQ or HCQ/AZ. **Panel J:** MFI of CD8 expression CD8^+^ CD44^+^TCF-1^+^ PD-1^+^ TILs in response to anti-PD-1 alone or in combination with HCQ or HCQ/AZ.

Given the effect of HCQ and AZ on CD8^+^ TILs, we next used flow cytometry combined with viSNE and Cytobank analysis to interrogate the composition of this subset (**Fig 3**). viSNE can visually define groupings of immune cells by simultaneously combining multiple markers [50]. Standard flow cytometric profiles showed that The CD8 compartment could be separated into different areas by staining with anti-PD-1 and anti-TCF-1 (**Fig 3A**). One island of CD8^+^ cells showed moderate to high levels of PD-1 and CD44 expression (island (i)), while a separate grouping of cells expressed primarily TCF-1 but no PD-1 (island (ii)). A grouping of cells between islands (i) and (ii) showed moderate levels of PD-1 and TCF-1 co-expression (island (iii)). TCF-1 defines progenitor CD8^+^ T cells [51, 52] which give rise to effector CD8^+^ T cells [35, 49].

We first assessed the presence of TCF-1^+^ PD-1^-^ and TCF-1^+^ PD-1^+^ progenitor TILs in response to anti-PD-1 therapy, either alone or in conjunction with HCQ or AZ. Anti-PD-1 did not alter the presence of CD8^+^ TCF-1^+^ PD-1^-^ cells relative to the overall CD8^+^ TIL population (**Fig 3B**) or relative to the tumor volume (**Fig 3C**). HCQ and HCQ/AZ also had no effect on the presence of this subset of TILs. By contrast, anti-PD-1 consistently increased the presence of CD8^+^ TCF1^+^ PD-1^+^ as measured relative to tumor volume (**Fig 3D**). Significantly, HCQ and HCQ/AZ inhibited this event. Similarly, anti-PD-1 therapy increased the presence of a subset of TCF1^+^ PD-1^+^ TILs expressing the receptor CD44 (**Fig 3E**). CD44 is a marker for antigen (Ag)-experienced, effector and memory T cells [53]. In this instance, we also compared the effects of HCQ and HCQ/AZ from tumors of responsive and non-responsive mice. Mice which were responsive to the effects of HCQ and HCQ/AZ had larger tumors (i.e. big), unaffected by anti-PD-1. By contrast, mice which were non-responsive to the effects of HCQ and HCQ/AZ had smaller tumors (i.e. small) similar in size to those seen with anti-PD-1 therapy alone. Tumors from responsive mice showed a clear inhibition of the presence of CD8^+^ TCF-1^+^ PD-1^+^ progenitors, while tumors from HCQ non-responsive mice showed an increase in the presence of progenitors similar to that seen with anti-PD-1 therapy.

In terms of receptor expression, dot plot profiles showed that anti-PD-1 increased the MFI for TCF-1 expression on CD8^+^ CD44^+^ TCF1^+^ TILs (**Fig 3F**). Co-therapy with HCQ and HCQ/AZ had marginal but significant effects on preventing anti-PD-1 from achieving statistical significance relative to untreated control mice. These data showed that anti-PD-1 increased both the numbers of CD8^+^ CD44^+^ TCF1^+^ cells as well as the level of TCF-1 expression on these cells and that these effects were inhibited by both HCQ and HCQ/AZ. Less obvious effects of HCQ on anti-PD-1 induced increases in PD-1 (**Fig 3G**), the transcription factor T-bet (**Fig 3H**) or the activation marker Ki67 were seen (**Fig 3I**). Interestingly, HCQ and HCQ/AZ increased the expression CD8 when treatment was combined with anti-PD-1 (**Fig 3J**). Overall, these data showed that HCQ has a specific effect in inhibiting the ability of anti-PD-1 immunotherapy to increase the generation of CD8^+^ PD-1^+^ TCF1^+^ and CD8^+^ CD44^+^ PD-1^+^ TCF1^+^ progenitor TILs relative to tumor volume.

### HCQ and AZ decreases the ability of anti-PD-1 to induce CD8^+^ effector TILs

**Fig 4:**
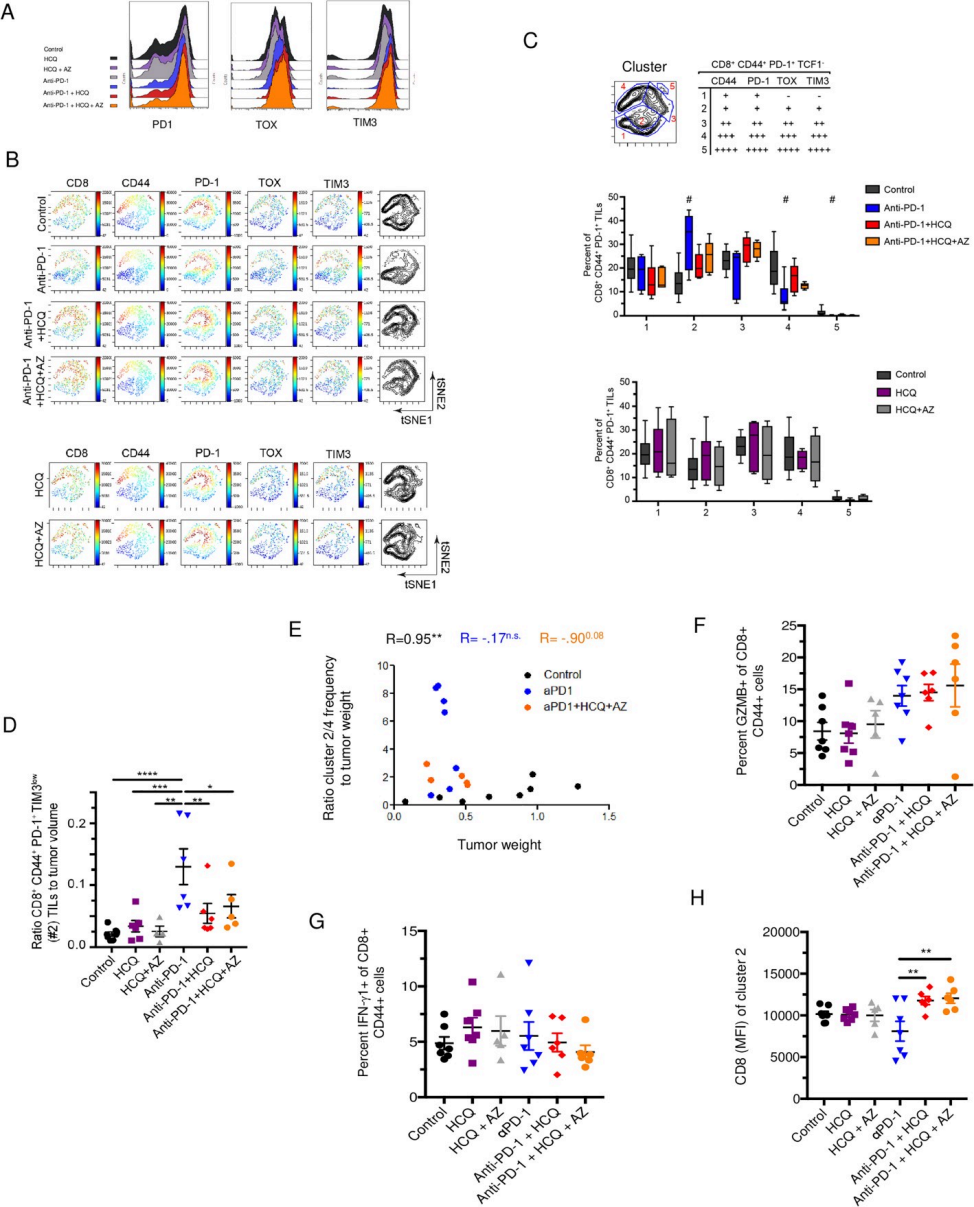
The expansion of specific CD8^+^TCF-1^-^ CD44^+^ PD-1^+^ subsets in response to anti-PD-1 is inhibited by HCQ and AZ. **Panel A:** Histogram profiles of anti-PD-1, TOX and TIM3 staining. **Panel B:** viSNE analysis of CD8, CD44, PD-1, TOX and TIM-3 expression of CD8^+^ CD44^+^ PD-1^+^ TILs. TCF1^+^ subsets have been removed. **Panel C:** tSNE subdivision into different clusters based on expression levels of CD44, PD-1, TOX and TIM3. Upper panel: Table shows how clusters were grouped together. Middle panel: Histogram showing changes in the representation of different clusters in response to anti-PD-1 alone or in combination with HCQ and AZ. Lower panel: Histogram showing changes in the representation of different clusters in response HCQ and HCQ/AZ. **Panel D:** Ratio of cluster 2 relative to tumor volume. Anti-PD-1 induced an increase in CD8^+^ effectors which was inhibited by HCQ and HCQ/AZ. **Panel E:** Figure showing the ratio of cluster2/4 to tumor weight. **Panel F:** Percent of GZMB^+^ cells within the CD8^+^ CD44^+^ PD-1^+^ TIL population. **Panel G:** Percent IFNγ^+^ cells within the CD8^+^ CD44^+^ PD-1^+^ TIL population. **Panel H:** CD8 expression (MFI) on cluster 2 TIL.

We next assessed the effect of HCQ and AZ on the generation of CD8^+^ effector TILs, which do not express TCF-1, but are derived from progenitors [54]. Standard flow profiles did not show an obvious difference in the presence of PD-1, TOX and TIM3 stained populations in response to various treatments (**Fig 4A**). By contrast, viSNE analysis was able to see clear differences in the presence of sub-populations of cells underscoring the power of the analysis (**Fig 4A**). 5 different clusters could be identified based on levels of receptor expression (**Fig 4B and 4C**). This included TCF1^-^ CD8^+^ cells with low-intermediate levels of PD1, TOX, TIM-3 (i.e. cluster 2: PD1^+^ TOX^+^ TIM-3^+^) as well as cells with higher levels of PD1, TOX, TIM-3 expression (i.e. clusters 3–5). Cells with the higher levels of PD-1 and TIM-3 expression classically correspond to exhausted T-cells [55, 56]. The status of cells with lower levels of these inhibitory receptors is unclear, but most likely are activated functional CD8^+^ T cells, which will eventually become exhausted, if exposed to repeated, ongoing antigenic stimulation.

From this, we observed that anti-PD-1 induced a clear increase in TCF1^-^ CD8^+^ cluster 2 expressing low levels of PD1, TOX, TIM-3 (i.e. PD1^+^TOX^+^TIM-3^+^) (upper table and middle panel). Significantly, HCQ and HCQ/AZ inhibited the increase in the presence of this population of cells. By contrast, no effect was seen on the presence of this population in anti-PD-1 untreated mice (lower panel). Dot plot analysis confirmed that anti-PD-1 increased the ratio of CD8^+^ CD44^+^ PD1^+^ TOX^+^ TIM-3^low^ TILs (cluster 2) relative to tumor volume and that this increase was blocked by the presence of HCQ or HCQ/AZ (**Fig 4D**). These data showed that HCQ inhibited the expansion of CD8^+^ TILs induced by anti-PD-1 therapy, expressing low levels of the activation markers PD-1 and TIM-3, either due to the inhibition of the presence of TCF-1^+^ PD-1^+^ progenitor T cells and/or due to direct effects on the PD1^+^ TOX^+^ TIM-3^+^ cells themselves.

At the same time, anti-PD-1 decreased the presence of CD8^+^ CD44^+^ T-cells with higher levels of PD-1, TIM-3 and TOX-3 corresponding to clusters 3–5 (**Fig 4B**). In the case of clusters 4 and 5, the decrease was statistically significant. HCQ and HCQ/AZ treatment inhibited the anti-PD-1 induced reduction in the presence of exhausted T-cells as most clearly seen in cluster 4. Unfortunately, as in other studies [57], due to small numbers of recovered TILs, it was not possible to assess directly whether these subsets of cells were functionally non-responsive or dysfunctional.

Importantly, an analysis of the cluster 2 (effector-like) to cluster 4 (terminal exhaustion) frequency ratio relative to tumor weight further showed that shift to cluster 2 in response to anti-PD-1 and its inhibition by HCQ/AZ was correlated with the loss of tumor control (**Fig 4D**).

Lastly, we observed that anti-PD-1 showed a trend in increasing the percentage of CD8^+^ CD44^+^ T cells expressing GZMB and this trend was unaffected by HCQ or HCQ/AZ (**Fig 4E**). Anti-PD-1 also showed a trend in increasing the percentage of CD8^+^ CD44^+^ T-cells expressing IFNγ which was unaffected by HCQ or HCQ/AZ (**Fig 4F**). Lastly, as was seen in the progenitor population, HCQ and HCQ/AZ had an usual effect on the expression of CD8 where it cooperated with anti-PD-1 to significantly increase the MFI of CD8 expression on the CD8^+^ effector-like (cluster 2) T-cells (**Fig 4G**).

Overall, these results showed that HCQ and HCQ/AZ interfered with the ability of anti-PD-1 to increase CD8^+^ effector-like TILs while at the same time prevented CD8^+^ TILs from acquiring a phenotype indicative of T-cell exhaustion. A general ability of HCQ to inhibit T cell activation would be consistent with this phenotype were it both inhibits the generation of functional CD8^+^ effectors while also preventing their progression into a more exhausted phenotype.

### HCQ and AZ did not affect antibody and vascular endothelial cells

**Fig 5:**
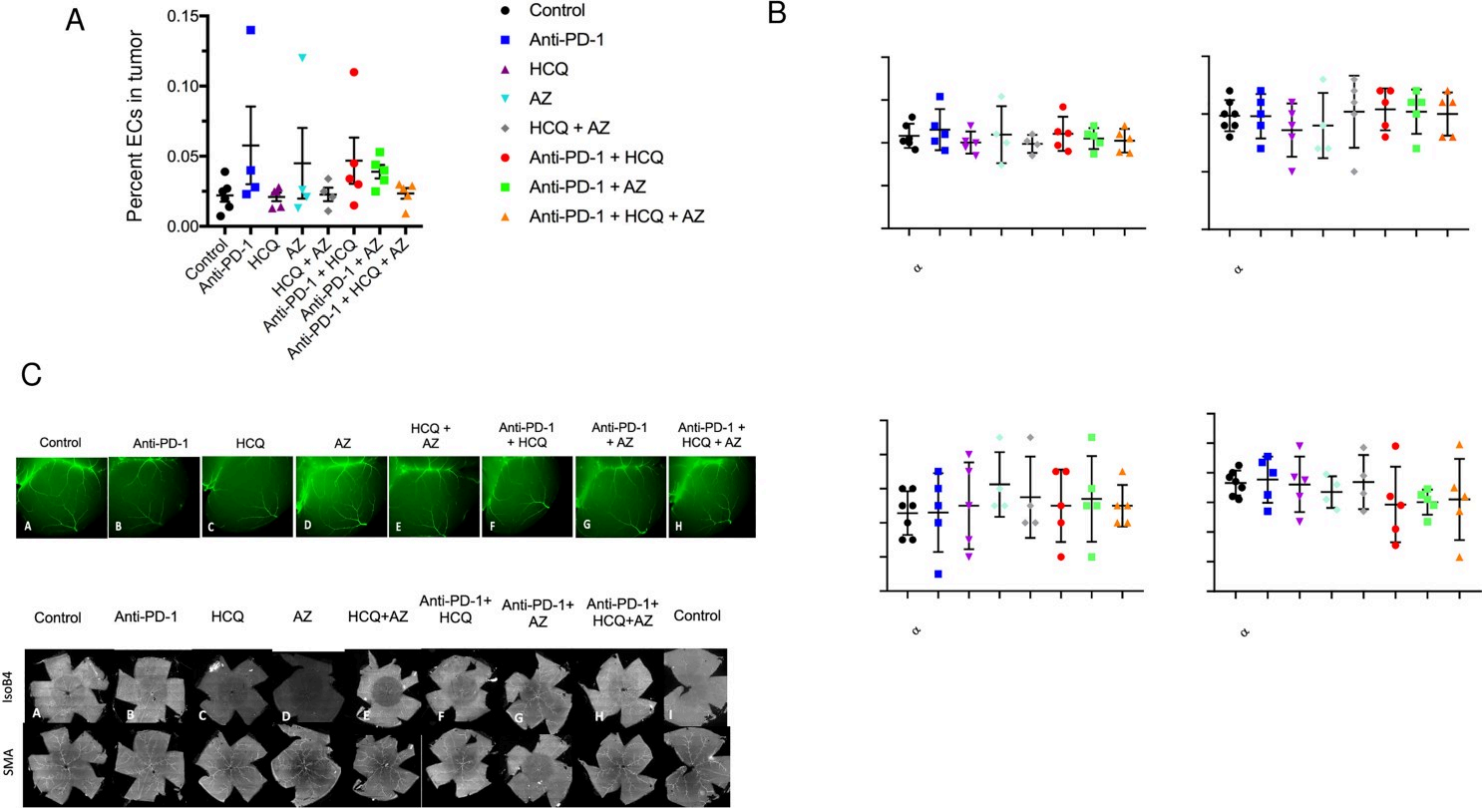
HCQ did not affect the vasculature. **Panel A:** Frequency of endothelial cells (ECs) relative to viable cells in the tumor. **Panel B:** Whole mount images of cerebral and retinal vessels. **Panel C:** Quantification of vessel bifurcation, arterial branches and vessel length in response to different treatments of mice [76].

Aside from the immune system, we also examined the vasculature in mice (**Fig 5**). To evaluate potential alterations in the morphological features of blood vessels, we examined retinal and brain cortical vasculature in treated mice. Whole mounts of retinal vasculature showed no changes in retinal vessel morphology or branching between groups. (**Fig 5A**). Further, an examination of the pial cerebral vessels, cortical vessels, vessel bifurcation and length showed no differences (**Fig 5B**). Images of retinal and brain cortical vasculature are shown in **Fig 5C**. These data indicate that neither anti-PD-1 nor HCQ or AZ had any detectable effects on the vasculature in mice. Furthermore, we found no effect of treatments on the overall branching structure in the cortical vasculature. These results show that the topological structure of the retinal and brain cortical vascular networks was not affected by the treatments.

## Discussion

HCQ continues to be used to treat thousands of patients with autoimmune disorders and infections with malaria and SARS-CoV-2. This situation makes an inquiry into the effects on HCQ and AZ on cancer immunotherapy an immediate pressing issue. In this study, we show that HCQ alone, or in combination with AZ, at the doses used to treat COVID-19 patients, reduces the ability of anti-PD-1 therapy to control the growth of cancer. This reduction was accompanied by a selective interference with CD8^+^ TCF1^+^ progenitor and CD8^+^ PD-1^+^ effector TIL expansion or infiltration of tumors. Further, we show that TCF-1 expression serves as a marker in peripheral blood CD8^+^ T cells for the response of the immune system to SARS-CoV-2 and HCQ therapy. The reduction in the presence of CD8^+^ subsets would account for the diminished anti-tumorigenic efficacy of PD-1 immunotherapy. Our study underscores the need for caution in the use of HCQ in the treatment of COVID-19 patients under immunotherapy.

We found that HCQ, either alone, or together with AZ, partially reversed anti-PD-1 immunotherapy in reducing the growth of B16 melanoma tumors in mice. The reversal was seen as early as days 12–16 post-implantation at therapeutic doses used to treat COVID-19 patients. No effect was observed on the growth of the B16 tumor in mice not treated with immunotherapy. Further, the effects were seen on the composition of infiltrating CD8^+^ T cells, and not on the growth of the tumor cells themselves. Mechanistically, HCQ and HCQ/AZ reduced an anti-PD-1 induced increase in the expression of PD-L1 on tumor cells, while exerting specific inhibitory effects on the presence of progenitor and effector CD8^+^ TILs. While the reduction in PD-L1 expression of B16 cells should have promoted tumor rejection, the concurrent inhibition of CD8^+^ progenitors and effectors appeared to dominant in the inhibition and reversal of the anti-PD-1 anti-tumor response. The targeting of CD8^+^ T cells by HCQ in anti-PD-1 treatment is consistent with the fact that CD8^+^ T cells are the prime target of immunotherapy elicited by anti-PD-1. We found no statistically significant effect on the presence of CD4^+^ T cells, B cells or myeloid cells. In this context, it is also noteworthy that T and B cells use different signaling pathways to initiate proliferation [58, 59]. Differences in signaling have also been noted between CD4^+^ and CD8^+^ T cells where IL-2 induces quantitatively stronger proliferation in CD8^+^ T cells compared to CD4^+^ T cells [60] due to greater IL-2Rβ chain expression [61]. We also failed to find differences in antibody titers in mice or in levels of IgG1/G2 antibodies against PD-L1. This susceptibility of CD8^+^ T-cells to inhibition by HCQ in the context of anti-PD-1 immunotherapy may also involve factors in the tumor microenvironment that affected immune activation and function.

As reported by others [57, 62], we observed that anti-PD-1 immunotherapy increased the presence of PD-1^+^ TCF-1^+^ progenitors and PD-1^+^ CD44^+^ TCF-1^-^ effector TILs. These progenitors in turn are needed to produce more differentiated PD-1^+^TCF1^-^ cells in immunotherapy [62]. We presented these changes in terms of percentages since the absolute numbers of TILs can decrease in small tumors and the subsets are connected to each other where, for example, TCF-1 renewing cells give rise to more mature T cells. Importantly, HCQ and HCQ/AZ impaired the ability of anti-PD-1 therapy to increase in the presence of these PD-1^+^ TCF-1^+^ progenitors. Consistent with this, HCQ also decreased the ability of anti-PD-1 to expand CD8^+^CD44^+^ TILs. These cells generally expressed low-intermediate levels of the activation markers PD-1 and TIM-3, and most likely represent a newly expanded effector population expressing these activation markers. However, co-expression of PD-1 and TIM-3 with TOX may also correspond to an early stage of cellular expansion leading to T cell exhaustion. In our model, anti-PD-1 therapy reduced the presence of CD8^+^ TILs with the high co-expression of PD-1, TIM-3 and TOX indicative of terminal exhaustion [63, 64]. Our study was able to distinguish between subsets with different levels of receptor expression showing the anti-PD-1 preferentially expands CD8^+^ effector cells with low-intermediate levels of PD-1, TIM-3 expression and actually limits the presence of more terminally exhausted CD8^+^ T cells. Differences in these findings could be related to the tumor type and the duration in anti-PD-1 treatment of mice. HCQ concurrently inhibited the anti-PD-1 induced reduction in this subset of terminally exhausted T cells, although the effect of the combination of HCQ/AZ did not inhibit the reduction, in the case of the CD8^+^ T cells with highest observed levels of PD-1, TIM3 and TOX indicative of the most severe exhaustion. Our observations are most consistent with a general ability of HCQ to inhibit the activation and proliferation of CD8^+^ TILs. It can inhibit the appearance of CD8^+^ progenitors, newly generated CD8^+^ effectors from progenitors and the progression of CD8^+^ effectors to a more exhausted phenotype.

Surprisingly, AZ alone failed to elicit a statistically significant effect in mice on tumor growth as observed for HCQ. AZ and ciprofloxacin (CPX) act as lipophilic weak bases where they affect intracellular organelles similar to CQ [46] and previous studies have documented a role for antibiotics such as ciprofloxacin (CPX) in the reversal of immune checkpoint blockade on tumour eradication [44, 45]. These previous studies emphasized effects on the gut microbiota [44, 45]. The differences in these findings may relate to the dose of antibiotics used or the coverage of bacterial inhibition by AZ versus ciprofloxacin (CPX) and other antibiotics. It might also relate to the nature of gut microbiota in mice which is likely to vary in different containment facilities.

In terms of the expression of specific molecules, HCQ had variable effects on markers indicative of activation. For example, while HCQ inhibited the expression of TCF1 TILs, it did not reproducibly affect the expression of the activation marker Ki67 in progenitor T-cells. One surprising observation was the potent effect of HCQ in increasing CD8 expression on progenitor and effector T-cells. It is paradoxical given our previous findings showing that CD8 (and CD4) bind to p56^lck^ to initiate a protein-tyrosine phosphorylation cascade in T cells that is needed for T cell activation [65, 66]. Further, the down-regulation of CD8-lck has been suggested as a mechanism for peripheral tolerance [67, 68], while subpopulations with low CD8 have been reported during chronic diseases [69] and in acute immune responses to pathogens [70]. HCQ and AZ increased CD8 expression in combination with anti-PD-1. No effect was seen in the absence of anti-PD-1 indicating that altered signaling linked to anti-PD-1 blockade synergizes with HCQ and AZ to promote increased CD8 expression. It was observed on progenitors and effector T cells. The functional consequences of this increased expression are unclear, although an increased number of CD8 molecules engaged by MHC during antigen-presentation would be expected to increase the activation and proliferation of T cells [59]. Alternatively, depending on the relative stoichiometry and localisation of expression, an excess of CD8 receptors might lead to few receptors bound to p56^lck^ and hence, reduced activation. CD4 sequestration of p56^lck^ complex may also prohibit the induction of activation signals through the TCR-CD3 complex [71].

Other studies have documented the benefits of HCQ on other aspects of anti-tumor immunity. Strikingly, Sharma et al. reported that HCQ potentiated the effects of anti-PD-1 against B16 melanoma cells [47]. The findings of this paper are at odds with the findings outlined in this paper. We used a lower HCQ concentration (daily 40 mg/kg) for 6 days while Armaravadi and colleagues injected daily 60 mg/kg for 11 days. Further, we started treatment with HCQ after detection of first signs of response to anti-PD-1 therapy. The tumor volume at that time-point was approximately 200 mm^3^. Sharma et al. started anti-PD-1 and HCQ treatment simultaneously and in 4x smaller tumors (50 mm^3^). Other possible explanations include a possible difference in tumor as we used a B16-PD-L1 tumor cell line, while Sharma inoculated B16.F10 cells. The Sharma paper also observed changes in macrophages and conditioned media. Myeloid cells plays a key role in anti-PD-1 immunity; however, responses ultimately depend on CD8^+^ T-cells which were seen to be defective in our model [49]. We would therefore argue that our findings extend the theme of immune suppression by identifying specific T-cell subpopulations in tumors responsible for this effect. Others have reported HCQ inhibition of CD4^+^ T cell activation [31], antigen-presentation [8, 33] and the production of proinflammatory Th17-related cytokines [34]. We have also outlined the potential benefits of targeting other mediators of T cell activation in the potentiation of anti-viral and tumor responses [72].

Despite this, we acknowledge that multiple immune cells play roles in orchestrating the immune response such that CQ can decrease the immunosuppressive infiltration of myeloid-derived suppressor cells and Treg cells [73] and HCQ inhibition of autophagy may synergize with dual anti-PD1 and anti-CTLA4 therapy [74]. The actions of HCQ may therefore be complex, with potential benefits and disadvantages dependent on the nature of anti-tumor immunology. However, our study signposts negative effects on T cells, the effectors of tumor immunity.

Many questions remain related to HCQ, COVID-19 and tumor immunology. Whether the inhibitory effects on anti-PD-1 immune therapy may be offset by its reported benefit in sensitizing chemotherapy remains to be determined. For example, HCQ has been reported to enhance the anti-cancer activity of the histone deacetylase inhibitor, vorinostat (VOR), in pre-clinical models and early phase clinical studies of metastatic colorectal cancer (mCRC) [75]. Lastly, the inhibitory effects on anti-PD-1 therapy in this study used HCQ doses that have been used in the treatment of COVID-19 patients [12–22]. It remains unclear whether lower concentrations of drug will have the same effect, although the effectiveness of lower concentrations in treating auto-immune diseases such as SLE is in part likely due to its immune-suppressive properties [8, 9]. The utility of HCQ may vary dependent on the dose of the drug and the nature of cancer therapy.

## Materials and methods

### Housing and ethical approval

C57BL/6 mice were housed at the Hôpital Maisonneuve Rosemont animal facility (Montreal, QC, Canada). Mice were housed in individually ventilated cages (IVC) and all experiments were approved by the CR-HMR Ethical Approval (Le Comité de protection des animaux du CIUSSS de l’Est-de-l’Île-de-Montréal (CPA-CEMTL), F06 CPA-21061 du projet 2017–1346, 2017-JA-001/2).

### Mice

C57BL/6 mice were housed at the Hôpital Maisonneuve Rosemont animal facility (Montreal, QC, Canada). Mice, aged 7–8 weeks, were implanted intradermally with 50,000 B16-PD-L1 melanoma cells that overexpress PD-L1. Cells were kindly provided by Dr. M. Ardolino, Ottawa, Canada. Anti-PD-1 treatment (200ug/mouse, i.p., 2x/week) started at day 4 post-implantation, when tumors were established. After 1-week of anti-PD-1 (clone: J43, BioXcell, West Lebanon, TX) treatment, first signs of tumor response were noted and HCQ (40 mg/kg, daily) and/or AZ (40 mg/kg for day 1 and 2; 20 mg/kg for day 3–5) were injected i.p. When giving combined, injections were performed within a 4 h time interval. Due to different pharmacokinetics of HCQ in mice, 40 mg/kg in mouse represents 600 mg in humans (Jackson et al., 2016, abstract, AACR). Tissues and PBMCs were harvested day 17 post-implantation.

### Chemicals

Hydroxychloroquine sulfate (HCQ) was purchased from Selleckchem and azithromycin (AZ) was purchased from Hemopharm (Vrsac, Serbia).

### Mouse tissue collection and preparation

Tumour infiltrating lymphocyte collection: Briefly, tumors were harvested and minced into small pieces before enzymatic digestion with Liberase TL (Sigma, Damstadt, Germany) and DNase1 (Thermo Fisher, Waltham, MA, USA) at 37°C for 30 min. Addition of RPMI with 10% FBS stopped reaction and mixture were passed through 70 μm cell strainers to generate single cell suspension. After 2 washes cells were (i) for total TILs quantification stained for viability and fixed or (ii) overlayed on a ficoll layer for lymphocyte collection. Lymphocytes were then stained for viability followed by surface staining with FACS antibodies before fixation. Intracellular staining was performed using the FoxP3/Transcription Factor Staining Buffer Set (ebiosciences, San Diego, CA, USA). For PBL isolation from mice, blood was collected in EDTA-coated tubes and peripheral blood mononuclear cells (PBMCs) were isolated by Ficoll (Corning, NY, USA). After 3 washes, PBMCs were stained for FACS. For the preparation of thymocytes, thymi were collected from all mice immediately after sacrifice, dissociated and passed through 70 μm cell strainers into RPMI medium. Following 3 washes, cells were stained for viability and fixed in 2% PFA until further analysis.

### Flow cytometry and antibodies

Antibodies were purchased from either Biolegend or BD Biosciences. Flow cytometric analyzes were performed according to standard protocols. In brief, PFA-fixed TILs were washed twice with cold PBS and stained with the targets listed below for 30 min on ice and then permeabilized for 45 min on ice. TILs were then stained for intracellular targets for 45 min on ice, and then washed twice with PBS and proceeded to data collection. The antibodies used as listed below:

Intracellular staining were performed using the FoxP3/Transcription Factor Staining Buffer Set (ebiosciences, San Diego, CA, USA). Cells were acquired using BD LSR Fortessa X-20 and DIVA software. FACS analyses were performed using FlowJo software and Cytobank premium. The antibodies were used to stain subsets of immune cells as outlined in the Table 1. All the markers used for immune population identification are described in Table 2.

**Table 1:** Antibody list used for cytometry staining.

Antibody	Flurochrome	Company
Live Dead	BV510	BD Bioscience
CD45	AF700	BioLegend
TCRb	BUV305	BD Bioscience
CD19	PE	BioLegend
CD11c	BV650	BioLegend
CD11b	APC	BioLegend
PD-L1	PeCP Cy5.5	BioLegend
CD31	PECy7	BD Bioscience
Ly6C	BV605	BioLegend
Ly6G	APC Cy7	BioLegend
MHC II	BV421	BD Bioscience
PD-1	BV786	BioLegend
CD80	FITC	BioLegend
CD8	BV650	BD Bioscience
CD44	BV605	BD Bioscience
CD62L	APC Cy7	BD Bioscience
TCF-1	APC	Cell Signaling
Tbet	PE Cy7	BioLegend
Ki67	PE	BioLegend
TOX	FITC	eBioscience
TIM3	PE	BioLegend
Granzyme B	eF450	eBioscience
IFNg	PE Dazzle	eBioscience

**Table 2:** Marker used for the identification of immune cell populations.

Cell population	Marker
Ecs	CD45- CD31+
B-cells	CD45+ CD19+ TCRb-
M-MDSCs	CD45+ CD11c- CD11b+ Ly6C+ Ly6G-
PMN-MDSCs	CD45+ CD11c- CD11b+ Ly6Clow Ly6G+
cDC	CD45+ CD11c+ MHC II+
DC1	CD45+ CD11c+ MHC II+ CD11b-
DC2 + DC3	CD45+ CD11c+ MHC II+ CD11b+
CD8+ TCM	CD45+ TCRb+ CD8+ CD4- CD44+ CD62L+
CD8 + TEM	CD45+ TCRb+ CD8+ CD4- CD44+ CD62L-
CD4 Thelper	CD45+ TCRb+ CD8- CD4+ FoxP3-
Regulatory T cells	CD45+ TCRb+ CD8- CD4+ FoxP3+

### Wholemount brain staining

Brains were fixed in 4% paraformaldehyde overnight at 4°C, washed 3 times in PBS, and blocked overnight in blocking solution (0.1Tris-HCl, 150 mM NaCl, 0.2% Blocking reagent (PerkinElmer), 0.5% Triton-X). After overnight incubation with anti-Smooth Muscle Actin-FITC (Sigma), brains were washed and imaged with the use of a fluorescent dissecting microscope.

### Retinal staining

Eyes were prefixed in 4% paraformaldehyde for 15 min at room temperature. Dissected retinas were blocked overnight at 4° C in blocking solution (0.1Tris-HCl, 150 mM NaCl, 0.2% Blocking reagent (PerkinElmer), 0.5% Triton-X). Retinas were then incubated with IsolectinB4 and immunostained with anti-Smooth Muscle Actin-FITC (Sigma). Quantification of retinal vasculature branch points was done with the use of the angiogenesis analyzer tool for ImageJ.

### Wholemount brain and retinal staining

Brains were fixed in 4% PFA overnight at 4°C, washed 3 times in PBS, and blocked overnight in blocking solution (0.1Tris-HCl, 150 mM NaCl, 0.2% Perkin Elmer blocking reagent and 0.5% Triton-X). After overnight incubation with anti-Smooth Muscle Actin-FITC (Sigma), brains were washed and imaged with the use of a fluorescent dissecting microscope.

Eyes were prefixed in 4% PFA for 15 min at room temperature. Dissected retinas were blocked overnight at 4°C in blocking solution (0.1Tris-HCl, 150 mM NaCl, 0.2% Perkin Elmer blocking reagent and, and 0.5% Triton-X). Retinas were then incubated with IsolectinB4 and immunostained with anti-Smooth Muscle Actin-FITC (Sigma). Quantification of retinal vasculature branch points was done using the angiogenesis analyzer tool for ImageJ.

### Anti-B16-PDL1 antibody assay

Plasma was collected from heparinized murine peripheral blood by centrifugation for 15 min at 2000g. Samples were immediately used or aliquoted and frozen at -80°C. For the antibody assay we used a slightly modified version of Dilillo D. et al. 2012 protocol. Briefly, 5x10^5^ B16-PDL1 tumour cells were added to a 96-well plated, blocked with 10% FBS-PBS, and washed with PBS. Plasma samples were diluted 1:16 in PBS and were added to B16 cells in a volume of 100 μl, mixed and then incubated for 1 hour at 4°C. Cells were intensively washed and secondary Goat anti-mouse IgG-af488 conjugated (A11029; Invitrogen) and Rat anti-mouse IgM-APC (clone RMM-1; Biolgend #406509) both at 1:500 dilution and incubated on ice for additional 1 hour. Cells were washed and passed to flow cytometry. **S5 Fig** Shows data from one experiment that is representative of all experiments held. Median fluorescence intensity (MFI) and the frequency of positively stained cells were assessed in FlowJo (Treestar). Index value was calculated by normalizing B16-IgG and B16-IgM MFI of the sample to the average of tumor-free mice.

### Statistics

All data are expressed as mean ± SEM. A t-test was used when only two groups were compared, and a one-way ANOVA was used when more than two groups were compared. A two-way ANOVA was used for multiple comparison procedures that involved two independent variables. Dunett or Bonferroni tests were used for *posthoc* analyses. A difference in mean values between groups was significant when p ≤ 0.05 *; p ≤ 0.01 ** and p ≤ 0.001 ***.

## Supporting information

S1 Fig Spider graphs representing the effects of HCQ and AZ on immune checkpoint blockade in cancer therapy. C57BL/6 mice were implanted with B16 tumoral cells as described above in Fig 1. **Panel A:** Tumor growths in control vs anti-PD-1 vs anti-PD-1 + HCQ vs anti-PD-1 + AZ vs anti-PD-1 + HCQ + AZ. **Panel B:** Spider graph representing control vs anti-PD-1. **Panel C:** Spider graph representing control vs anti-PD-1 + HCQ. **Panel D:** Spider graph representing control vs anti-PD-1 + HCQ + AZ. **Panel E:** Spider graph representing control vs anti-PD-1 + AZ.
(PDF)

S2 Fig Spider graphs representing the effects of HCQ and AZ in control groups. C57BL/6 mice were implanted with B16 tumoral cells as described above in Fig 1. **Panel A:** Tumor growths in control vs HCQ vs AZ vs HCQ + AZ. **Panel B:** Spider graph representing control vs HCQ + AZ. **Panel C:** Spider graph representing control vs HCQ. **Panel D:** Spider graph representing control vs AZ.
(PDF)

S3 Fig Correlation between tumor wright and tumor volume in response to treatments. **Panel A:** Anti-PD-1. **Panel B:** Anti-PD-1 + HCQ. **Panel C:** Anti-PD-1 + HCQ/AZ.
(PDF)

S4 Fig HCQ and AZ do not inhibit the in vitro growth of B16 melanoma cells. **Left panel:** Viability of B16 cells after a 24 incubation with 2 and 5uM AZ or HCQ or in combination as determined by trypan blue exclusion. **Right panel:** Viability and metabolism of B16 cells as determined by an MTT assay. The MTT assay is a colorimetric assay for assessing cell metabolic activity. NAD (P)H-dependent cellular oxidoreductase enzymes can also reflect the number of viable cells.
(PDF)

S5 Fig The effect of HCQ and AZ on the production of subclasses of immunoglobulin against PD L-1. Serum IG levels were measured in response to anti-PD-1 and HCQ or AZ. **Panel A**: IgM levels (MFI). **Panel B**: IgG levels (MFI). **Panel C**: IgG levels (%). **Panel D**: IgG levels (index value). **Panel E**: IgM and IgG (median index value). **Panel F**: IgM (index value).
(PDF)

S6 Fig The effect of HCQ ad AZ on the cellularity of thymic subsets. **Panel A:** CD4 MFI on CD4+ thymocytes. **Panel B:** CD8 MFI on CD8+ thymocytes. **Panel C:** CD4 MFI on double positive (CD4 and CD8+) DP thymic subsets. **Panel D:** CD8 MFI on double positive (CD4 and CD8+) DP thymic subsets. **Panel E:** Percent representation of thymic subsets in response to anti-PD-1, HCQ and HCQ/AZ. **Panel F:** Medium size representation of thymic subsets in response to anti-PD-1, HCQ and HCQ/AZ. **Panel G:** HCQ uptake on thymocytes.
(PDF)
