# Supplementary material for: Hydroxychloroquine (HCQ) decreases the benefit of anti-PD-1 immune checkpoint blockade in tumor immunotherapy
Source: PLoS One. 2021 Jun 28;16(6):e0251731. doi: 10.1371/journal.pone.0251731 (PMC8238207; doi:10.1371/journal.pone.0251731)
Supplement: S1 Fig — C57BL/6 mice were implanted with B16 tumoral cells as described above in Fig 1. Panel A: Tumor growths in control vs anti-PD-1 vs anti-PD-1 + HCQ vs anti-PD-1 + AZ vs anti-PD-1 + HCQ + AZ. Panel B: Spider graph representing control vs anti-PD-1. Panel C: Spider graph representing control vs anti-PD-1 + HCQ. Panel D: Spider graph representing control vs anti-PD-1 + HCQ + AZ. Panel E: Spider graph representing control vs anti-PD-1 + AZ. (PDF) [file pone.0251731.s001.pdf]

Figure S1

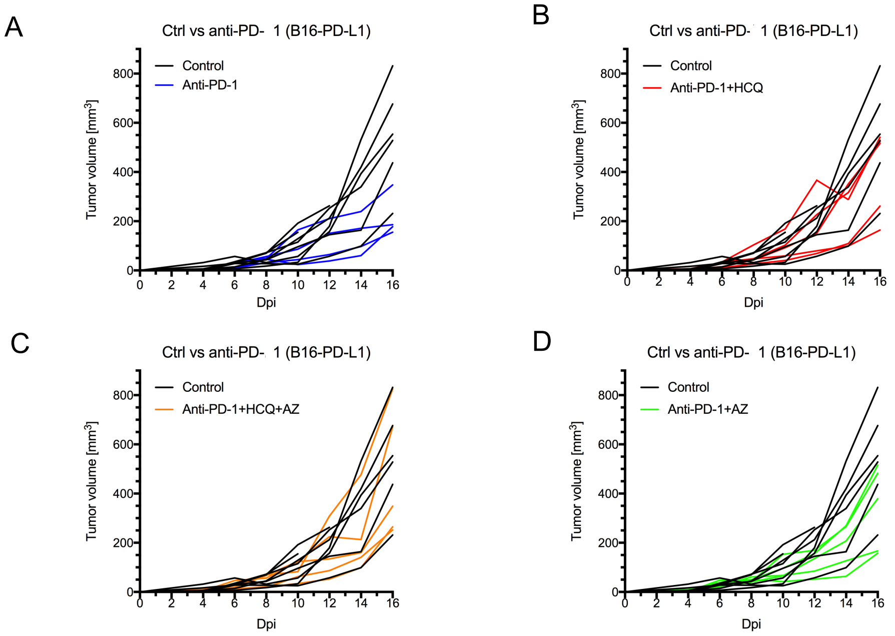

Figure S1: Spider graphs representing the effects of HCQ and AZ on immune checkpoint blockade in cancer therapy. C57BL/6 mice were implanted with B16 tumoral cells as described above in Figure 1.
Panel A: Tumor growths in control vs anti-PD-1 vs anti-PD-1 + HCQ vs anti-PD-1 + AZ vs anti-PD-1 + HCQ + AZ.
Panel B: Spider graph representing control vs anti-PD-1.
Panel C: Spider graph representing control vs anti-PD-1 + HCQ.
Panel D: Spider graph representing control vs anti-PD-1 + HCQ + AZ.
Panel E: Spider graph representing control vs anti-PD-1 + AZ.
