# Supplementary material for: Hydroxychloroquine (HCQ) decreases the benefit of anti-PD-1 immune checkpoint blockade in tumor immunotherapy
Source: PLoS One. 2021 Jun 28;16(6):e0251731. doi: 10.1371/journal.pone.0251731 (PMC8238207; doi:10.1371/journal.pone.0251731)
Supplement: S2 Fig — C57BL/6 mice were implanted with B16 tumoral cells as described above in Fig 1. Panel A: Tumor growths in control vs HCQ vs AZ vs HCQ + AZ. Panel B: Spider graph representing control vs HCQ + AZ. Panel C: Spider graph representing control vs HCQ. Panel D: Spider graph representing control vs AZ. (PDF) [file pone.0251731.s002.pdf]

Figure S2

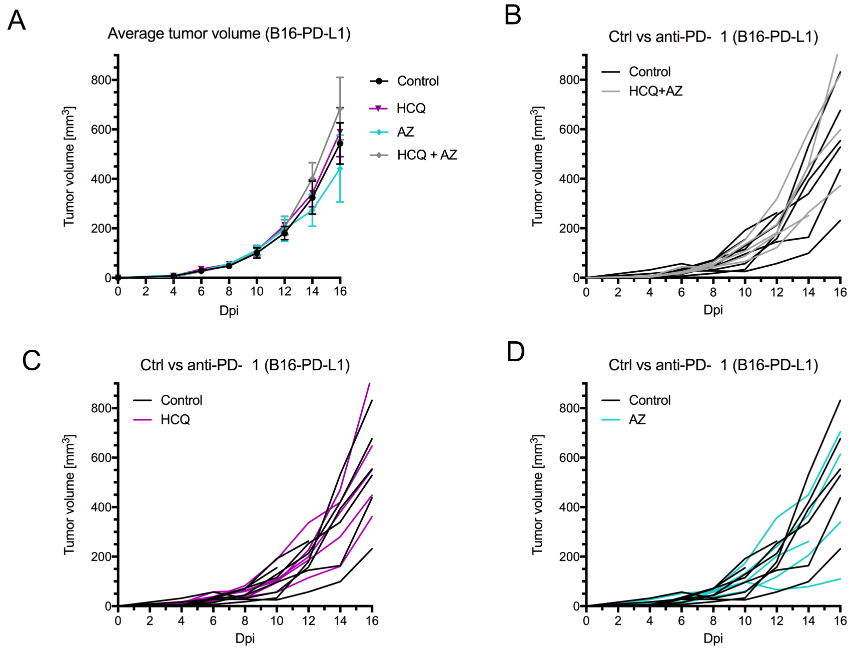

Figure S2: Spider graphs representing the effects of HCQ and AZ in control groups. C57BL/6 mice were implanted with B16 tumoral cells as described above in Figure 1.
Panel A: Tumor growths in control vs HCQ vs AZ vs HCQ + AZ.
Panel B: Spider graph representing control vs HCQ + AZ.
Panel C: Spider graph representing control vs HCQ.
Panel D: Spider graph representing control vs AZ.
