# Supplementary material for: Hydroxychloroquine (HCQ) decreases the benefit of anti-PD-1 immune checkpoint blockade in tumor immunotherapy
Source: PLoS One. 2021 Jun 28;16(6):e0251731. doi: 10.1371/journal.pone.0251731 (PMC8238207; doi:10.1371/journal.pone.0251731)
Supplement: S3 Fig — Panel A: Anti-PD-1. Panel B: Anti-PD-1 + HCQ. Panel C: Anti-PD-1 + HCQ/AZ. (PDF) [file pone.0251731.s003.pdf]

Figure S3

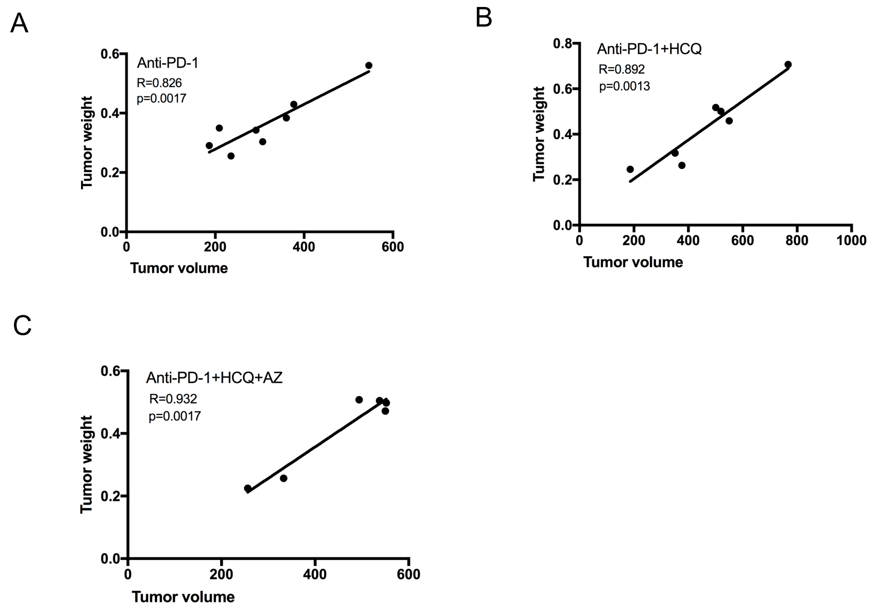

Figure S3: Correlation between tumor wright and tumor volume in response to treatments
Panel A: Anti-PD-1
Panel B: Anti-PD-1 + HCQ
Panel C: Anti-PD-1 + HCQ/AZ
