# Supplementary material for: Hydroxychloroquine (HCQ) decreases the benefit of anti-PD-1 immune checkpoint blockade in tumor immunotherapy
Source: PLoS One. 2021 Jun 28;16(6):e0251731. doi: 10.1371/journal.pone.0251731 (PMC8238207; doi:10.1371/journal.pone.0251731)
Supplement: S4 Fig — Left panel: Viability of B16 cells after a 24 incubation with 2 and 5uM AZ or HCQ or in combination as determined by trypan blue exclusion. Right panel: Viability and metabolism of B16 cells as determined by an MTT assay. The MTT assay is a colorimetric assay for assessing cell metabolic activity. NAD (P)H-dependent cellular oxidoreductase enzymes can also reflect the number of viable cells. (PDF) [file pone.0251731.s004.pdf]

Figure S4

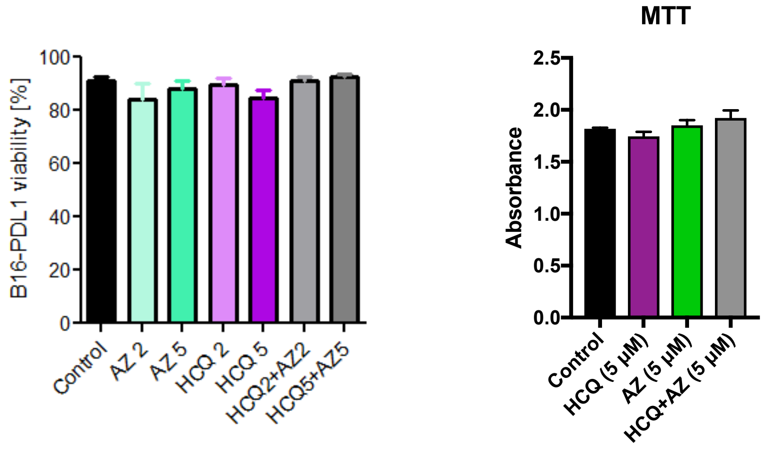

Figure S4: HCQ and AZ do not inhibit the in vitro growth of B16 melanoma cells

Left panel: Viability of B16 cells after a 24 incubation with 2 and 5uM AZ or HCQ or in combination as determined by trypan blue exclusion.
Right panel: Viability and metabolism of B16 cells as determined by an MTT assay. The MTT assay is a colorimetric assay for assessing cell metabolic activity. NAD (P)H-dependent cellular oxidoreductase enzymes can also reflect the number of viable cells.
