# Supplementary material for: Hydroxychloroquine (HCQ) decreases the benefit of anti-PD-1 immune checkpoint blockade in tumor immunotherapy
Source: PLoS One. 2021 Jun 28;16(6):e0251731. doi: 10.1371/journal.pone.0251731 (PMC8238207; doi:10.1371/journal.pone.0251731)
Supplement: S5 Fig — Serum IG levels were measured in response to anti-PD-1 and HCQ or AZ. Panel A: IgM levels (MFI). Panel B: IgG levels (MFI). Panel C: IgG levels (%). Panel D: IgG levels (index value). Panel E: IgM and IgG (median index value). Panel F: IgM (index value). (PDF) [file pone.0251731.s005.pdf]

Figure S5

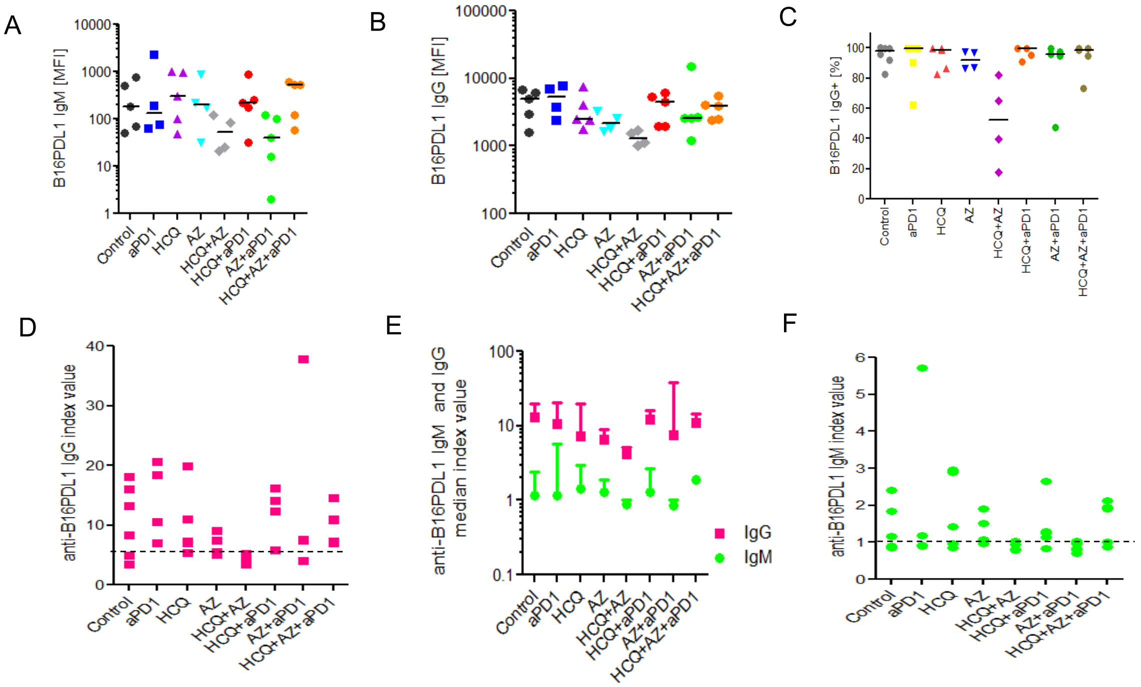

Figure S5: The effect of HCQ and AZ on the production of subclasses of immunoglobulin against PD L-1. Serum IG levels were measured in response to anti-PD-1 and HCQ or AZ.

Panel A: IgM levels (MFI)
Panel B: IgG levels (MFI)
Panel C: IgG levels (%)
Panel D: IgG levels (index value)
Panel E: IgM and IgG (median index value)
Panel F: IgM (index value).
