# Supplementary material for: Hydroxychloroquine (HCQ) decreases the benefit of anti-PD-1 immune checkpoint blockade in tumor immunotherapy
Source: PLoS One. 2021 Jun 28;16(6):e0251731. doi: 10.1371/journal.pone.0251731 (PMC8238207; doi:10.1371/journal.pone.0251731)
Supplement: S6 Fig — Panel A: CD4 MFI on CD4+ thymocytes. Panel B: CD8 MFI on CD8+ thymocytes. Panel C: CD4 MFI on double positive (CD4 and CD8+) DP thymic subsets. Panel D: CD8 MFI on double positive (CD4 and CD8+) DP thymic subsets. Panel E: Percent representation of thymic subsets in response to anti-PD-1, HCQ and HCQ/AZ. Panel F: Medium size representation of thymic subsets in response to anti-PD-1, HCQ and HCQ/AZ. Panel G: HCQ uptake on thymocytes. (PDF) [file pone.0251731.s006.pdf]

Figure S6

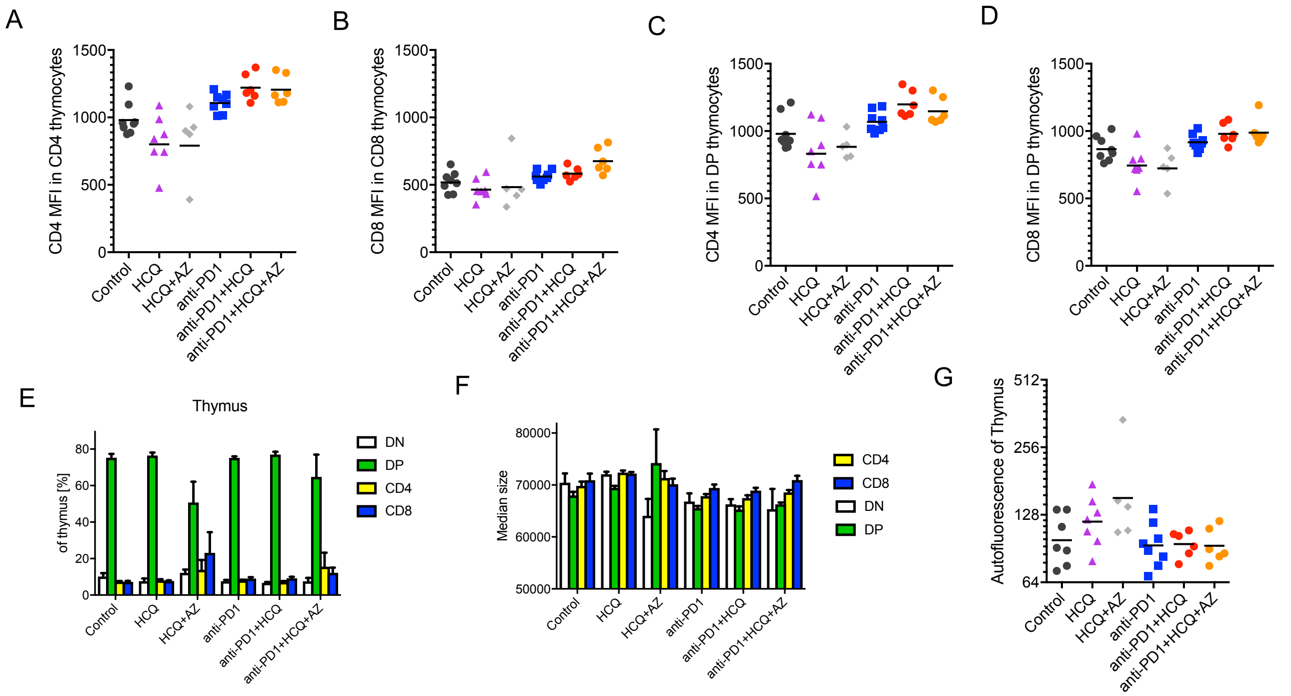

Figure S6: The effect of HCQ ad AZ on the cellularity of thymic subsets
Panel A: CD4 MFI on CD4+ thymocytes
Panel B: CD8 MFI on CD8+ thymocytes
Panel C: CD4 MFI on double positive (CD4 and CD8+) DP thymic subsets
Panel D: CD8 MFI on double positive (CD4 and CD8+) DP thymic subsets
Panel E: Percent representation of thymic subsets in response to anti-PD-1, HCQ and HCQ/AZ
Panel F: Medium size representation of thymic subsets in response to anti-PD-1, HCQ and HCQ/AZ
Panel G: HCQ uptake on thymocytes
